# Denosumab for osteoporosis treatment: when, how, for whom, and for how long. A pragmatical approach

**DOI:** 10.1007/s40520-025-02991-z

**Published:** 2025-03-08

**Authors:** Olivier Lamy, Judith Everts-Graber, Elena Gonzalez Rodriguez

**Affiliations:** 1 Department of Medicine, Internal Medicine, Lausanne University Hospital and University of Lausanne, Lausanne, Switzerland; 2 Interdisciplinary Center of Bone Diseases, Rheumatology Unit, Bone and Joint Department, Lausanne University Hospital and University of Lausanne, Lausanne, Switzerland; 3 OsteoRheuma Bern, Bahnhofplatz 1, Bern, Switzerland; 4 Department of Rheumatology and Immunology, University Hospital, Bern, Switzerland; 5 Service de médecine interne, CHUV, Rue du Bugnon 46, Lausanne, 1011 Switzerland

**Keywords:** Denosumab, Osteoporosis, Rebound effect, Fracture, Multiple vertebral fractures

## Abstract

Denosumab produces a continuous increase in bone mineral density over ten years, associated with a low risk of vertebral and non-vertebral fractures. Denosumab is well tolerated and easy to manage in daily clinical practice. For all these reasons, this treatment has a huge success. On the other hand, discontinuation of treatment is associated with a severe rebound effect including a sharp increase in bone turnover markers, loss of the bone density gained and a risk of nearly 20% of multiple vertebral fractures in postmenopausal women. High doses of potent bisphosphonates are needed to maintain bone turnover markers in the low range of premenopausal women, to mitigate this rebound effect. Prolonged treatment with denosumab is associated with a greater rebound effect and increases the risk of an early rebound effect. The occurrence of rare side effects such as osteonecrosis of the jaw or atypical femoral fracture, as well as the onset of severe renal failure, leave clinicians at a therapeutic impasse. Continuing denosumab or switching to bisphosphonates remains suboptimal and, currently, no evidence clarifies the optimal treatment approach for these patients. The aim of this review is to give a very practical clinical approach to the use of denosumab (duration of treatment), and to the management of rebound effect and possible adverse effects.

## Introduction

Denosumab, a monoclonal antibody against the receptor activator of nuclear factor κ-Β ligand (RANKL), is a powerful antiresorptive agent. Denosumab was approved by European and US regulatory authorities in 2010. The dose approved for osteoporosis is 60 mg administered subcutaneously every 6 months. In the 10-year FREEDOM Extension study, treatment with denosumab resulted in a continuous increase in bone mineral density (BMD), associated with a low risk of vertebral and non-vertebral fractures (VFs) [1]. The treatment is very well tolerated, with very few side effects. Because of its ease of use, safety profile and continued efficacy over the years, this treatment has been a huge success. This has led some experts to propose long or very long-term treatment. Strategies such as “treat to target” or targeting a total hip T-score of -1.5 have been developed to minimize the risk of fractures [2]. However, these results and the strategies derived from them do not consider the rebound effect after denosumab discontinuation, or the occurrence of adverse events. The risk of osteonecrosis of the jaw (ONJ), atypical femoral fracture (AFF), or how to manage discontinuation in case of severe renal failure leave clinicians at a therapeutic dead end.

In the absence of bisphosphonate relay, discontinuation of denosumab results in a rebound in osteoclasts activity, characterized by an increase in bone turnover markers (BTMs), the loss of the BMD gained, and an increased risk of spontaneous VF probably close to 20% in postmenopausal women [3–5]. Despite bisphosphonate therapy, some of the BMD gained is lost [3, 6, 7]. The longer denosumab is taken, the greater the loss and the greater the risk of multiple VFs [6, 8, 9]. It is therefore necessary to find a compromise between a gain in BMD associated with a significant reduction in fracture risk, and a limited rebound effect enabling part of the benefit to be preserved. The occurrence of osteonecrosis of the jaw (ONJ) or atypical femoral fracture (AFF) constitutes a therapeutic dead-end [10]. These complications require denosumab to be discontinued. As bisphosphonates are contraindicated, patients are exposed to a rebound effect with a high risk of multiple VFs. However, some authors suggest continuing antiresorptive treatment despite the presence of an ONJ or AFF or resuming antiresorptive treatment after healing/operation of the primary lesion. The occurrence of chronic kidney disease stage IV or V is another therapeutic dead-end. On denosumab, these patients may experience severe and prolonged hypocalcemia, which can be life-threatening [11–13]. In addition, bisphosphonates are contraindicated, particularly at doses required to manage the rebound effect [14].

There are no randomized controlled trials to answer the difficult questions mentioned above. A few clinicians have tried to come up with original solutions to manage the rebound effect and unusual situations. The aim of this article is to offer a pragmatic approach to denosumab management and discontinuation.

## For which patient (Table 1)

**Table 1:** The key points before and during denosumab treatment

1. Ensure patient (and family) compliance and manage injection dates at the doctor’s office
2. A broad biological work-up to exclude a secondary cause of osteoporosis
3. Injections should be given strictly every 6 months (+/- 2 weeks). Do not exceed 6 months for treatments lasting more than 3 years.
4. Dose CTX once a year on the day of denosumab injection. CTX must be very low. If they rise to 50% of the upper limit of the norm for premenopausal women, think of an early rebound.
5. Perform DXA every 2 years
6. Anticipate denosumab discontinuation with bisphosphonate therapy

Denosumab is recommended for men and postmenopausal women with osteoporosis who are at high risk of fractures [15, 16]. It is also a treatment option for glucocorticoid-induced osteoporosis [17], and for patients undergoing hormone-ablative therapies, such as those with early breast cancer receiving aromatase inhibitors or non-metastatic prostate cancer undergoing androgen deprivation therapy [18, 19]. Denosumab is well-tolerated and easy to manage in daily clinical practice. It is effective as both a first-line treatment and as a second-line option following bisphosphonates or anabolic agents [20–22]. Despite its facility of use, the key question is how to integrate denosumab into a long-term treatment strategy that may span several decades and involve multiple treatment sequences in patients whose comorbidities that will evolve over the years.

Strict adherence to the 6-month dosing schedule is crucial, as delays are associated with bone loss and an increased risk of VFs [23], similarly to the rebound effect seen after discontinuation. Delayed treatment is associated with a fourfold increased risk of VFs [24]. This is particularly relevant for older, frail patients, who may forget appointments, be hospitalised or experience changes in their care team, such as moving to a nursing home. Therefore, when initiating denosumab, both the patient (and/or their relatives) and all involved healthcare providers must be fully informed of the importance of maintaining strict dosing intervals to ensure patient safety. Thus, patients with pathologies that do not guarantee optimal compliance, not only during denosumab treatment, but also during the management of the rebound effect, are not good candidates for this treatment.

Precautions should be taken when starting denosumab in patients with renal insufficiency or at risk of developing renal insufficiency. Furthermore, denosumab is not appropriated in patients with renal insufficiency and low bone turnover. These patients are at high risk of hypocalcemia or severe hypocalcemia requiring hospitalization [11, 12]. In a retrospective study of dialysed patients, 607 of 1523 denosumab-treated patients and 23 of 1281 oral bisphosphonate-treated patients developed severe hypocalcemia (41.1% vs. 2.0%) [13]. Management of hypocalcemia involves ensuring normal blood levels of 25-OH vitamin D and administering high doses of calcium and calcitriol. It may also be necessary to use dialysis baths with higher calcium levels. In January 2024, the FDA added a boxed warning about the increased risk of severe hypocalcemia in patients with advanced chronic kidney disease taking denosumab, including an increased risk of life-threatening illness or death. In addition, the high doses of bisphosphonates needed to manage the rebound effect after denosumab discontinuation are contraindicated in patients with renal failure. If denosumab is prescribed to a patient with or at risk of worsening renal impairment, treatment should be of short duration as it eases rebound control (see below). It must be ensured that high doses of bisphosphonates can be administered at the end of treatment without impairing renal function.

In clinical practice, discontinuing denosumab and switching to bisphosphonates is often approached with caution. This is especially true for younger patients and those on long-term denosumab treatment, both of which are associated with greater bone loss after transitioning to bisphosphonates [6, 7]. Denosumab is not contraindicated in younger patients. However, it should be reserved for 2nd-line treatment after failure of a bisphosphonate, or at first line for less than 3 years to reduce the severity of the rebound effect (see below). One could argue that denosumab should be administered indefinitely. Indeed, it has been tested for up to 10 years, demonstrating both efficacy and safety over this period, and possibly even longer [1]. There are currently no data on the effects of such prolonged suppression of bone remodelling, but concerns exist regarding potential long-term adverse events, such as osteonecrosis of the jaw or atypical femoral fractures. Thus, while lifelong denosumab could be an option for some older patients, it may not be the optimal strategy for younger individuals due to the unknown risks of extended use.

Another question is whether denosumab has a place in the prevention of bone loss (as opposed to established osteoporosis). This concerns women and men without osteoporosis who are receiving hormone-ablative therapies. Since managing the rebound effect after denosumab discontinuation is challenging, it would appear to be an excessive step for a preventive treatment. Moreover, in some countries there may be concerns on the management of denosumab discontinuation as potent bisphosphonates are not reimbursed in the absence of osteoporosis.

## Bisphosphonate before denosumab?

The benefit of a treatment with a potent bisphosphonate (alendronate, zoledronate) after denosumab to avoid BTMs increase, BMD loss and the risk of spontaneous VFs, is well established. As bisphosphonates have a prolonged residual effect on the bone matrix, giving them before denosumab could also help limiting the rebound after denosumab discontinuation. Studies of bisphosphonate use prior to denosumab have several limitations, including retrospective design, small sample size, lack of timeline on bisphosphonate initiation and treatment duration. Moreover, these studies only included patients who had not received a bisphosphonate after denosumab, and thus covered a period prior to the recommendations for post-denosumab bisphosphonate [25]. Two small series analyzing the effect on BTMs showed a reduction in the increase in BTMs during the rebound effect [26, 27]. Small series show no benefit in terms of BMD loss reduction during the rebound effect. One series demonstrated that higher BMD loss after denosumab discontinuation was associated with lack of prior antiresorptive treatment [6]. Several studies have found no protective effect against spontaneous VFs [25]. Only one large study involving 797 women followed for up to 30 months after the last denosumab injection demonstrated that a bisphosphonate given before denosumab offered partial protection against VFs during the rebound effect, but did not provide any additional benefit if a bisphosphonate was given after denosumab [3]. This observation is particularly important, not for patients who decide to stop treatment (since they will receive a bisphosphonate), but for those who risk delaying an injection, thereby increasing their risk of vertebral fractures.

Considering all these results, it seems possible that bisphosphonate prior to denosumab attenuate the hyperactivation of BTMs and may reduce the risk of VFs in case of injections delay.

## Treatment duration

The FREEDOM trial, which tested denosumab against placebo, demonstrated safety and efficacy for 3 years [28]. The FREEDOM extension trial showed that continuing denosumab for another 7 years lead to continued BMD gain and further fracture reduction, which is unique compared to bisphosphonates [1]. Both alendronate and zoledronic acid usually show a plateau after 5 and 3 years, respectively [29, 30]. It is unclear why denosumab leads to continued BMD gains beyond 5 years, when resorption cavities are filled, and bone turnover remains suppressed. One hypothesis, though not proven, suggests a potential anabolic effect with long-term use [31], but the evidence supporting this remains limited [32].

Treat-to-target strategies in osteoporosis management have been proposed since 2013, with fracture prevention being the primary goal and the attainment of higher BMD levels as the most optimistic surrogate target [33, 34]. Many randomised controlled trials with anti-osteoporotic agents demonstrated that larger increases in BMD are associated with greater reduction in fracture risk, whereas achieving T-scores > -2.5 to -2.0 with treatment appears to provide little additional fracture protection. For denosumab therapy, targeting a total hip T-score between around − 1.5 to -2.0 is associated with a reduction of non-VFs [2]. Therefore, because of the continuous increase in BMD with longer treatment duration, denosumab is often considered an optimal option for treat-to-target therapy approaches. However, the challenge lies in maintaining this “safe haven”. Indeed, the BMD loss associated with the rebound after switching from denosumab to bisphosphonates is more pronounced following long-term (10 ± 2 vs. 5 ± 2 injections) denosumab therapy [35], and in the FREEDOM and FREEDOM Extension Trials denosumab duration was significantly associated with multiple VFx (> 3 years vs. *≤* 3 years; odds ratio 3.0; 95% CI, 1.4–6.5) [9]. The attenuation of BTMs reduction at the end of the 6-month interval between two doses of denosumab decreases with time: -72.1% after the first dose, -47.5% after the eighth dose [36]. Longer denosumab treatment has been associated with earlier development of VFs [37]. Indeed, VFs may occur as early as 6 or 7 months after the last denosumab injection [3, 8]. These latter considerations suggest that an early rebound may occur with the longer duration of denosumab treatment. It is therefore crucial that the treatment is carried out strictly every 6 months, and without delay, especially if the treatment is of long duration. While greater BMD gains can be achieved with prolonged denosumab use, the potential for significant loss afterwards increases too, and the final BMD gain may be close in both cases [35, 38]. This must be carefully considered when planning sequential treatment regimens for patients. The choice of treatment should be based on the lowest baseline T score, which enables more than 50% of women to achieve a T score > -2.5 in around 3 years. For example, for denosumab: -3.1 DS at the lumbar spine, and − 2.8 DS at the total hip [39].

### Short versus long-term denosumab therapy (Table 2)

**Table 2:** Denosumab: clinical consequences related to the number of doses

**One dose**
No rebound effect.
As emergency (acute vertebral fracture) and decide 6 or 7 months later which treatment to use.
One or two doses followed by a bisphosphonate give good BMD results.
**Two to six doses**
The rebound effect is less severe.
**More than six doses**
The rebound effect is more severe.
The risk of missing a dose or of a delay of more than six months between two doses is increased.
Risk of early rebound effect (after 5 months).
Risk of multiple vertebral fractures during the rebound effect increases with duration of treatment.

A single dose of denosumab does not induce a rebound effect [27]. This is particularly useful for the clinical management of denosumab. For example, a patient who takes denosumab once and experiences side effects can stop treatment. It is possible to administer one dose of denosumab in an emergency (for example, in the event of an acute VF) and to decide, after a full assessment, to continue after 6 to 7 months with another antiresorptive or anabolic treatment.

While there is no single ‘optimal’ duration for denosumab therapy, treatment should be individualized based on the patient’s specific situation. Older individuals, or those with very low bone density and a high fracture risk, may benefit from lifelong denosumab therapy, provided adherence can be ensured. In contrast, for younger patients below 75–80 years, a shorter treatment duration (e.g., less than three years) may be more appropriate to mitigate the risk of a significant rebound effect. Interestingly, evidence suggests that after one or two denosumab injections, no excessive bone resorption occurs [27]. Therefore, to completely avoid rebound, one option is to administer a single or two denosumab injections followed by a switch to bisphosphonates, which has been shown to result in substantial BMD gains [35, 38]. However, in high-risk patients, denosumab should not be switched to a bisphosphonate too early, as low bone density at the time of transition increases the risk of rebound-associated vertebral fractures [3]. That’s why, in younger high-risk patients, initial treatment should ideally be anabolic, aiming to significantly improve BMD and reduce the imminent fracture risk before switching to an antiresorptive therapy. Denosumab first-line may be suboptimal in this context.

## How to manage denosumab discontinuation

When discontinuing denosumab, the main objective is to avoid VFs secondary to the rebound effect. Avoid BMD loss to keep the benefit obtained under denosumab is also a primary objective. Several facts have been noted concerning the management of denosumab discontinuation after randomized-controlled studies [20, 40, 41] and observational studies in clinical routine conditions [6, 8, 37, 42, 43].

- Only potent bisphosphonates (alendronate and zoledronate), in many cases at high doses, seem to be effective to control the rebound effect, at least partially [3, 6, 41, 44, 45]. Zoledronate after a 2 to 5-year denosumab treatment seem to be not associated with multiple VFs [45]. However, alendronate has been studied mainly after short-term treatment with denosumab (1 to 2 years) [20, 44].
- Reports comparing their effects with less potent antiresorptive or anabolic agents show that these are not sufficiently efficacious in preventing bone loss or VFs. Transitioning to raloxifene and risedronate induces almost a complete loss of the BMD gained with denosumab [5, 38, 46]. A transient bone loss was seen with teriparatide [20]. The transient bone loss was more pronounced at cortical sites. Total hip and femoral neck BMD increased more in the teriparatide to denosumab group than in the denosumab to teriparatide group: + 6.6% vs. + 2·8% (total hip) and + 8·3% vs. 4·9% (femoral neck). Romosozumab loses 50–100% of its efficacy when administered after denosumab [47]. After 12 months of treatment with romosozumab, changes in lumbar spine BMD were + 18.2% in previously untreated patients and + 6.4% in patients previously treated with denosumab. At the femoral neck, the BMD changes were + 4.9% and + 0.7% respectively. Moreover, after transitioning to raloxifene 23% presented with VFs [5] and case reports describe rebound VFs despite risedronate, romosozumab, but also alendronate treatment [40, 47, 48].
- Rebound intensity is stronger with longer denosumab treatment, as shown by increased risk of multiple VFs without subsequent treatment [9]. Other risk factors were identified to influence the magnitude of the rebound effect: prevalent vertebral fractures, greater gain in hip BMD during therapy, and greater loss of hip BMD off therapy [52]. Bone loss increased despite the switch to bisphosphonates in patients who received more than 3 years of denosumab compared with those who received shorter treatment [38, 49, 50].
- Whatever the bisphosphonates dosage at denosumab discontinuation, some patients will lose BMD, and this loss is associated to increased levels of BTMs [42, 43].
- Because multiple VFs can occur as soon as 6–7 months after last denosumab injection [3, 51], transitioning to bisphosphonates should not be delayed. Also, delaying bisphosphonates relay is associated to higher BMD loss [52].

It has been shown that, to avoid most VFs at denosumab discontinuation, a simple protocol is probably sufficient: a single zoledronate perfusion 6 months after last denosumab injection [6, 51]. However, due to the before described facts, the optimal treatment regimen to avoid bone loss has not yet been established, although practical guidelines applicable in most countries have been proposed in 2020 by the ECTS [53]. We thus describe here our personal approach and usual practice derived from our clinical experience.

1. To slow down the onset of the rebound, weekly oral alendronate can be introduced 4 months after the last denosumab injection (Table 3). As osteoclasts start resorbing bone, it will progressively bind to hydroxyapatite and block their activity. In the event of poor compliance with alendronate, zoledronate will be indispensable approximately 6 months after the last denosumab.
2. Measure fasting CTX (alternatively, if patients are not fasting, P1NP can be used with caution due to a slight delay compared to CTX) every 1–2 months thereafter. Continue with alendronate if CTX is in the lower 60% of the upper limit of the normal values for premenopausal women (i.e. 400 ng/L for an upper limit of 600 ng/L). Measure fasting CTX every two months. Perform a 5 mg zoledronate infusion as soon as they rise over 60% of the upper limit of the normal values.
3. Follow fasting CTX every 3 months after first zoledronate infusion during the first year of the rebound, and every 6 months during the second year. Follow BMD yearly.
4. Repeat the zoledronate infusion if fasting CTX increase over the target, or if there is a clinically significant BMD loss (over the least significant change for the machine).

**Table 3:** Management of denosumab discontinuation with alendronate

1. Start Alendronate 4 months after the last denosumab injection.
2. Measure CTX 7 months after last denosumab injection.
3a If CTX are low*, continue alendronate and measure CTX every two months.
3b If CTX are in the target**, switch to zoledronate iv and measure CTX every three months
3c If CTX are above the normal values for premenopausal women, repeat a new denosumab injection, and start again at point 1, advancing the various proposals by 1 month.
4. Each time CTX is above the optimal target***, repeat zoledronate iv.
5. If DXA 18 months after the last denosumab show a significant bone loss, repeat zoledronate

* low: < 20% of the upper limit of the normal value for premenopausal women

** between 20% and 100% of the normal value for premenopausal women (if CTX are slightly increased, Alendronate 2x/week may be an option)

*** below the 60% of the normal values for premenopausal women

In our experience, up to 5 perfusions may be needed over the 2 years to maintain low bone remodelling. No randomized trial has provided evidence that repeated zoledronate infusions can prevent rebound-associated bone loss, and some observational studies have also not demonstrated a benefit from additional infusions [41, 43, 49]. However, no alternative exists to mitigate the increase in bone turnover, except restarting denosumab. It should be noted that there is no description of atypical fractures or ONJ development during the rebound period treated with high zoledronate cumulative doses, probably due to the presence of high remodelling driving the indication for repeated doses.

Alternatively, these options can be considered:

- In patients with short denosumab treatment (< 3 years), or renal insufficiency (down to 25 ml/min/1,73m^2^), alendronate can be continued for up to 24 months after last denosumab injection (instead of relay by zoledronate), with CTX follow-up every 2 months during the first 6 months, and every 4 to 6 months thereafter (Table 3). A 5 mg zoledronate perfusion, or an increase in alendronate dosing in renal insufficiency patients (twice weekly alendronate) should be given if fasting CTX increase over the target, or if there is a clinically significant BMD loss.
- Target CTX values can be lower in high-risk patients, i.e. 280 ng/L as proposed in the ECTS guidelines, which have been associated with no bone loss if maintained over the rebound period [42, 43]. In the study published by Grassi et al., CTX levels > 212 ng/L had 100% sensitivity for identifying patients with worsened BMD at follow-up and might therefore be the cut-off to aim during the follow-up to obtain BMD stability [42]. However, this can be difficult to achieve.
- If bone remodelling markers are not available, start alendronate 4 months after the last denosumab injection, and infuse zoledronate 5 mg 7 months and 12 months after the last denosumab injection (Table 4). Repeat treatment in the event of clinically significant loss of BMD at the lumbar spine or total hip densitometry, 1 and/or 2 years after the end of denosumab efficacy.

**Table 4:** Management of denosumab discontinuation with zoledronate

**A. Without CTX measurement**
1. Start Alendronate 4 months after the last denosumab injection.
2. Give iv Zoledronate 7 and 12 months after the last denosumab injection.
3. If DXA 18 months after the last denosumab show a significant bone loss, repeat zoledronate
**B. With CTX measurement**
1. Measure CTX 6 months after the last denosumab injection.
2a If CTX are low*, measure it again every month until it reaches the target value**.
2b If CTX are above the normal values for premenopausal women, repeat the denosumab injection and repeat the above regimen at 5 months instead of 6 months.
2c If CTX are within target**, give zoledronate
3. measure CTX every three months. Each time CTX is above the optimal target***, repeat zoledronate iv.
4. If DXA 18 months after the last denosumab show a significant bone loss, repeat
zoledronate

* low: < 20% of the upper limit of the normal values for premenopausal women

** between 20% and 100% of the normal values for premenopausal women

*** below the 60% of the normal values for premenopausal women

Finally, weaning denosumab with progressively lower doses is considered an alternative. In the phase 2 trial, denosumab 30 mg every 6 months or every 3 months, or denosumab 14 mg every 3 months had the same efficacy on BMD as alendronate [54]. But denosumab 14 mg every 6 months was less effective than alendronate. In the latter case, the “rebound effect” on BTMs was observed after 3 months, and at 6 months, i.e. before the next injection of 14 mg, BTMS were at the same level as at the beginning of treatment. A dose of 14 mg of denosumab should therefore be administered every 3 months. In one study, 114 women on denosumab 60 mg every 6 months were switched to denosumab 30 mg every 6 months [55]. After two years, BMD at the hip and radial sites was stable, and showed a small improvement at the lumbar spine. However, this study does not answer the question of denosumab discontinuation. In another study, 13 patients received six months after the last dose of denosumab 60 mg, a reduced dose of 30 mg, and, at month 12, a 15 mg injection [56]. A BMD performed one year after the last injection shows that this protocol does not prevent bone loss at the hip and partially maintains the initial gain at the spine. In addition, one patient presented two VFs, 8 months after the last dose of denosumab 15 mg. This study shows that a one-year tapering scheme does not cover the entire 24-month rebound period. Furthermore, a dose of 15 mg is not effective as long as 6 months [54]. In our experience (OL, EG), the inhibition of BTMs lasts 6 months with a 30 mg injection of denosumab. With a 15 mg injection, this inhibition lasts from 4 to 6 months, depending on the patient. When bisphosphonates are contraindicated or refused by a patient, we give denosumab 15 mg injections every 4 months for 2 years, with a BMD measurement every year. The results show a stable BMD. In some patients, denosumab 15 mg can be discontinued after 2 to 3 years. For the majority, however, treatment with bisphosphonates is necessary, but at doses lower than those used to manage rebound after 60 mg doses. Attitude is guided by CTX monitoring. In these cases, we tolerate that CTX are at the upper normal range for premenopausal women (i.e. normal values for patients). We propose a dose of denosumab 15 mg every 4 months in cases of contraindication to bisphosphonates or refusal by the patient.

### Vertebral fractures at denosumab discontinuation

The main risk factors for rebound-associated VFs are longer duration of denosumab treatment, length of time off denosumab treatment, prevalent VFs, greater loss of BMD after denosumab discontinuation, and, depending on the studies, younger (severity of the rebound) or older (missed denosumab doses) age [57]. The risk of VFs after denosumab discontinuation is associated with an increased risk of non-VFs [3]. There is no relevant data on the optimal treatment in patients suffering from rebound VFs, with only some published case reports. While in osteoporotic VFs an anabolic treatment is recommended, these are contraindicated in the case of rebound VFs because of the further increase in bone remodelling markers observed after denosumab discontinuation with teriparatide and romosozumab [20, 47]. Moreover, two case reports showed the development of new VFs up to 6 months after romosozumab introduction [58, 59]. Although relapse of VFs has also been described after denosumab restoration [60, 61]. This happened in a much shorter time (1–3 months) suggesting full effect had not been obtained. It has also been clearly demonstrated that new VFs develop in case of vertebroplasties [8, 51], which are thus contraindicated. We thus propose restoration of denosumab for at least one year, which can be combined with teriparatide in patients with high number of VFs or low bone densitometry.

## ONJ and AFF

Osteonecrosis of the jaw (ONJ) and atypical femoral fractures (AFF) are rare but serious side effects associated with anti-resorptive therapies. Their incidence increases with the duration of treatment. These side effects are well known with bisphosphonates. Their incidence with denosumab is less clearly documented. Based on the US FDA adverse event reporting system, the strongest significant signals showed that ONJ and atypical femur fracture could be more frequent with denosumab than with zoledronate [10].

### ONJ

The incidence of bisphosphonates-related ONJ in osteoporosis is around 2.5 per 10,000 patient-years in national registers [62, 63]. The incidence of ONJ during treatment with denosumab (60 mg every 6 months) is not well known. In the 7-year extension of the FREEDOM study, 13 patients developed ONJ, representing an incidence of 5.2 per 10,000 patient-years [1]. In patients with malignant diseases who received high doses of antiresorptive therapies, the risk of ONJ was higher in patients receiving denosumab (0.5 to 2.1% after 1 year, 1.1 to 3.0% after 2 years, and 1.3 to 3.2% after 3 years) compared with zoledronate (0.4 to 1.6%, 0.8 to 2.1%, and 1.0 to 2.3%, respectively) [64]. In a prospective cohort study of patients having dental extractions, the risk of ONJ in patients on denosumab for osteoporosis was 7.7 times more likely than patients treated with oral bisphosphonates [65]. Interestingly, the 76 patients who had dental extractions between the 6th and 7th month after the last denosumab injection did not develop ONJ. A registry-based study in Switzerland found a 3.4-fold increased risk of ONJ in osteoporosis patients treated with denosumab compared to those on bisphosphonates, particularly among denosumab patients with prior bisphosphonate use [66]. This may, at least in part, be explained by the total cumulative dose of antiresorptive therapies, which is likely higher in patients undergoing sequential treatments.

If ONJ occurs during antiresorptive treatment, the drug should be discontinued. This is not a problem with bisphosphonates. However, stopping denosumab exposes the patient to a rebound effect with a risk of multiple VFs. Relaying denosumab with high doses of bisphosphonates to manage the rebound effect is not recommended, as recovery from ONJ may be compromised. On the other hand, healing of ONJ associated with denosumab appears to be more rapid than bisphosphonate-related ONJ, possibly because bisphosphonates may impair healing not only of bone but also of soft tissues [67].

Continuing denosumab (dose 60 mg) is an option, but there too, the recovery from ONJ may be compromised. The alternative proposed by the authors (OL, EG) is to give a small dose of denosumab (15 mg = 1/4 dose) at shorter intervals (every 4 months). We have used this regimen on several occasions, with a favourable evolution of ONJ each time. If dental procedures are planned under denosumab, the authors (OL, EG) suggest that they are carried out 4–5 months after the last denosumab injection. In case of delayed healing at the time next injection is planned, a small dose of denosumab (15 mg) can be given instead. The alternative of performing dental procedures 6 months after previous injection (at the end of denosumab efficacy) and delaying next one can be potentially dangerous, moreover as some patients may experience an early rebound effect as early as 5 months after the last denosumab treatment.

### AFF

The incidence of AFF in patients receiving BP ranges from 0.6 per 10,000 patient-years to 13.1 per 10,000 patient-years depending on the duration of treatment (< 3 years or > 8 years) [68]. Less is known about AFF in patients receiving denosumab. AFF with denosumab has been described in several case reports and in three randomised controlled trials, but seem to be rare [1, 69, 70]. In the US FDA adverse event reporting system, AFF is more frequent with denosumab than with zoledronate [10].

In the presence of thigh or groin pain, AFF should be sought. In the case of unilateral AFF, the contralateral side should always be assessed. Conservative treatment is an option only for patients with incomplete fractures. Intramedullary nailing is the treatment of choice for most authors in both complete and incomplete AFF. Teriparatide appears to be the most attractive drug option for accelerating healing in AFF [71]. Discontinuation of antiresorptive therapy following AFF is strongly recommended, particularly to prevent contralateral AFF and to facilitate fracture healing. If an AFF occurs during BP therapy, BP can be discontinued. In contrast to BPs, discontinuation of denosumab without subsequent antiresorptive therapy may result in a rebound effect with an increased risk of multiple VFs. If denosumab is continued, the risk of contralateral AFF seem to be increased [72]. A short course of BP or treatment with SERM or teriparatide after denosumab discontinuation are proposed by some authors [73]. However, the risk of multiple VFs could be increased due to insufficient control of the osteoclast activity [5]. The alternative proposed by the authors (OL, EG) is to give a small dose of denosumab (15 mg) every 4 months. We have used this regimen successfully in the case of conservative treatment of AFF without the development of contralateral AFF. We have not yet published these cases.

## Conclusion

Denosumab is a valuable treatment in the osteoporosis therapeutic arsenal. The initial popularity of this treatment has been overshadowed by the multiple vertebral fractures occurring at its discontinuation. While denosumab is easy to administer, managing its discontinuation remains difficult. Moreover, there is no consensus. When treatment is stopped, a decrease in the bone density gained must be accepted in any case. Ultimately, this treatment raises more questions and problems than it answers: what is the optimal duration of treatment (< 3 years)? how should rare side effects be managed? what to do in case of renal failure? what treatment regimen should be used to manage the rebound effect?

As long as these questions remain unanswered, denosumab remains a second-choice treatment and should not be used to prevent bone loss.

## Data Availability

No datasets were generated or analysed during the current study.
